# Physicians' Perceptions of Barriers and Facilitators to the Improvement of Healthcare Equity for Children Hospitalized With Traumatic Brain Injury: Preliminary Findings From a Pilot Multicenter Pediatric Trauma Study From the United States

**DOI:** 10.7759/cureus.81036

**Published:** 2025-03-23

**Authors:** Chelsea D Hicks, Heather Barnett, Jennifer Shi, Julia Velonjara, Mauricio A Escobar, Darci Evans, John Fisher, Arnett Klugh III, Katrina M Morgan, Morgan K Richards, Sarah Risen, Courtney Robertson, Irim Salik, Dennis W Simon, Arul S Thirumoorthi, Deidre L Wyrick, Bryan J Weiner, Theresa J Hoeft, Monica S Vavilala

**Affiliations:** 1 Pediatrics, University of Washington School of Medicine, Seattle, USA; 2 Harborview Injury Prevention and Research Center, University of Washington, Seattle, USA; 3 Environmental Health Services, Public Health – Seattle and King County, Seattle, USA; 4 Rehabilitation Medicine, University of Washington School of Medicine, Seattle, USA; 5 Anesthesiology and Pain Medicine, University of Washington School of Medicine, Seattle, USA; 6 Pediatric Surgery and Pediatric Trauma, Mary Bridge Children's Hospital, Tacoma, USA; 7 Pediatric Critical Care Medicine, Harbor-University of California Los Angeles Medical Center, Los Angeles, USA; 8 Pediatric Trauma, Maria Fareri Children's Hospital-Westchester Medical Center, Valhalla, USA; 9 Neurosurgery, University of Nebraska Medical Center, Omaha, USA; 10 General Surgery, University of Pittsburgh Medical Center, Pittsburgh, USA; 11 Pediatric Surgery, St. Luke's Children's Hospital, Boise, USA; 12 Pediatric Neurology, Baylor College of Medicine, Houston, USA; 13 Anesthesiology and Critical Care, Johns Hopkins University School of Medicine, Baltimore, USA; 14 Anesthesia, Westchester Medical Center, Valhalla, USA; 15 Pediatric Critical Care Medicine, University of Pittsburgh Medical Center, Pittsburgh, USA; 16 Pediatric Surgery, University of Michigan, Ann Arbor, USA; 17 Pediatric Surgery, Arkansas Children's Hospital, Little Rock, USA; 18 Global Health, University of Washington, Seattle, USA; 19 Psychiatry and Behavioral Sciences, University of Washington, Seattle, USA

**Keywords:** critical care, equity, healthcare, pediatrics, traumatic brain injury

## Abstract

Background and objective

Disparities in outcomes for pediatric patients with traumatic brain injuries (TBI) in rural populations and for racial and ethnic minority groups have been documented. In light of this, we examined physician champions' perceptions of healthcare equity for hospitalized children with TBI.

Methods

We surveyed 10 physician TBI champions at 10 US pediatric trauma centers (PTCs) regarding organizational characteristics, barriers, and facilitators (domains and specific) in terms of improving healthcare equity, and priorities to redress inequities.

Results

Level I center TBI champions reported more pediatric beds and higher staffing-to-patient ratios while Level II TBI champions reported more pediatric TBI transfers. Across PTCs, the leading specific barriers were lack of access to post-discharge services, lack of staff training, and inadequate staffing. Level I PTCs identified a lack of knowledge about resources while Level II centers identified low hospital staffing numbers and lack of staff training as specific barriers. Across all PTCs, the leading specific facilitators were providers being up to date on skills, treatments, continuing education, team structure and cohesion, and quality improvement and protocol implementation. Across all PTCs, priorities to address barrier domains were staffing, cost and supply constraints, and organizational and structural domains, whereas priorities for facilitator domains were staffing, organizational and structural, and culture of change with variation in priority ranking to address barriers and facilitators by PTC level type. Physician champions identified common and unique barriers and facilitators to providing equitable healthcare for children hospitalized with TBI by PTC level type.

Conclusions

Respondents across all PTCs reported a set of common leading specific barriers and facilitators. Level I and Level II PTCs reported common specific barriers but more variable specific facilitators. Across all PTCs, the most frequently reported barrier domains were not always of the highest priority to redress.

## Introduction

Traumatic brain injury (TBI) is a leading cause of death and disability among children in the US [1]. Short- and long-term outcomes after pediatric TBI are worse in rural populations and among racial and ethnic minority groups [2-11]. African American, Hispanic, and Native American communities experience longer hospital length of stay (LOS), and girls may have higher TBI-related medical complications than boys [6]. The disparities in pediatric TBI outcomes may be attributed to inequities in TBI healthcare delivery related to organizational, structural, and process factors. While disparities are documented for TBI incidence and outcomes [6-8], there is scarce data on disparities in healthcare delivery during hospitalization for pediatric TBI.

Published literature suggests that challenges associated with the optimization of healthcare equity for TBI outcomes may include upstream structural barriers such as lack of funding for trauma centers where children receive acute care, lack of sufficient interpreters to provide language access, lack of training for caregivers of children with TBI, lack of provider and/or systems understanding of how social determinants of health affect TBI care, and/or lack of coordinated care [3-9]. However, the deployment of health equity champions in clinical fields may be an important intervention to support the prioritization of healthcare equity [12]. The Brain Trauma Foundation guidelines do not address strategies to decrease disparities during acute severe pediatric TBI critical care [13].

Given the widespread inequities in pediatric TBI, the study aimed to gain a preliminary understanding of the perspectives of pediatric TBI physician champions on barriers and facilitators to improving healthcare equity for children hospitalized with TBI across and by pediatric trauma center (PTC) level.

## Materials and methods

The University of Washington Institutional Review Board deemed this study exempt (STUDY00016161). The survey was administered starting in September 2022 until full participation was achieved. Participants' agreed sites would not be kept anonymous since the participants themselves were study authors.

Setting

A convenience sample of 11 Level I and Level II US PTCs were invited to participate based on the following criteria: (1) representation from Northeast, South, Midwest, and West census regions; (2) representation of rural and urban PTCs; (3) inclusion of Level I and II PTCs; (4) location in census areas serving diverse patient populations; and (5) response to email invitation regarding the willingness to participate. We contacted known trauma or TBI directors to identify physician pediatric TBI champions and included sites with diversity in geography, rural and urban status, and census data of populations served by the hospital; collectively, we intended for sites to represent a spectrum of hospitals caring for children with TBI.

Participants

Each site identified their physician TBI champion as follows: from any medical specialty, with leadership, scholarly activity, research expertise, and/or educational program development in pediatric TBI, and serving in leadership roles and as points of contact in equity improvement initiatives.

Study design

We administered a 35-item prospective cross-sectional survey organized into four sections: (1) site organizational characteristics (checkboxes and short answer), (2) perceived barriers and facilitators to equity in acute pediatric TBI care (checkboxes), (3) priority ranking of barrier and facilitator domains to reduce healthcare disparities (ranked list), and (4) clarifying responses (free text).

Survey development was informed by the Health Equity Implementation Framework and the Consolidated Framework for Implementation Research to understand common structural, organizational, and cultural factors that either facilitate or create barriers to improving healthcare equity [10-12,14-20]. These frameworks and existing literature informed the development and grouping of barriers and facilitator domains [21-24]. The survey did not define equity but asked respondents to reflect on the meaning of this term to answer the survey.

Domains

Table 1 shows the list of perceived barrier and facilitator domains and associated specific barriers and facilitators addressed in the survey. Each of the seven domains was separated into specific barriers and facilitators. Barriers were defined by “lack of or missing” a characteristic, and facilitators were defined by “presence” of a characteristic. The survey asked participants to select barriers and facilitators to equitable pediatric TBI care present at their center and rank priorities for addressing barriers and facilitator domains. Additionally, a free text option invited participants to share reasons for ranking barrier and facilitator domains and to suggest additional specific barriers and facilitators not in the survey list.

**Table 1:** Survey on perceived barriers and facilitators to equitable pediatric TBI care Notes: no additional context was provided to questions TBI: traumatic brain injury

Barriers and facilitators: equitable pediatric TBI care	
Pediatric TBI is a leading cause of death and disability in the US and worldwide. Disparities in acute adult TBI care are observed, recognized, and reported but such data in pediatric TBI are limited. Short- and long-term health effects of pediatric TBI are worse for populations based on geographic location and identifying as racial/ethnic minorities. **We acknowledge that barriers to functioning and care exist at every level of care within centers; however, we specifically want you to think from the perspective of barriers and facilitators for equitable pediatric TBI care.	
Barriers	Facilitators
Below, please select BARRIERS (any obstacle that limits or prevents people from receiving equitable care) to equitable pediatric TBI care at your center. (Check all that apply)	Below, please select FACILITATORS (the presence of elements that assist in equitable care) for equitable pediatric TBI care at your center. (Check all that apply)
1. Organizational and structural domain	1. Organizational and structural domain
Poor communication structures (e.g., between colleagues, management, and departments)	Good communication structures (e.g., between colleagues, management, and departments)
Transitions/handoffs	Transitions/handoffs
Team structure/cohesion	Team structure/cohesion
Physical environment/space (e.g., availability of beds)	Physical environment/space (e.g., availability of beds)
Priorities of leadership	Priorities of leadership
N/A	N/A
2. Knowledge and resources domain	2. Knowledge and resources domain
Providers have difficulty remaining up to date on skills/treatments/continuing education	Providers remain up to date on skills/treatments/continuing education
Limited knowledge about resources available for better care	Extensive knowledge about resources available for better care
Training of less experienced or new staff	Robust training/continuing education opportunities
N/A	Training of less experienced or new staff
	N/A
3. Cost and supply constraints domain	3. Cost and supply domain
Insurance status (uninsured or underinsured)	Insurance status (e.g., high insurance coverage)
Out-of-pocket costs	Few out-of-pocket costs
Patients' ability to access services upon discharge (e.g., travel distance/time to health center, lack of transportation)	Patients' ability to access services upon discharge (e.g., travel distance/time to health center, availability of transportation)
Limited availability of rehab services in the community	Availability of rehab services in the community
Limited availability of transportation services to your center	Access to transportation
N/A	N/A
4. Time constraints domain	4. Time efficiencies domain
High demand/pace of work (e.g., pressure of patient load)	Manageable demand/pace of work
Scheduling delays	Efficient scheduling system
Flexibility of work (e.g., lack of autonomy to prioritize tasks)	Flexibility of work (e.g., autonomy to prioritize tasks)
Missing workplace technologies	Availability of workplace technologies
N/A	N/A
5. Staffing domain	5. Staffing domain
Low staffing (numbers)	High staffing (numbers)
Staffing type/expertise	Staffing type/expertise
Staff (doctors, nurses, other support services) attitudes/motivation/ownership	Staff attitudes/motivation/ownership
High staff turnover	Low staff turnover
Reliance or presence of a large number of temporary or traveling nursing staff	More experienced staff
N/A	Ability to recruit and hire temporary nursing staff
	N/A
6. Culturally relevant factors domain	6. Culturally relevant factors domain
Lack of staff (doctors, nurses, other support services) diversity	Staff (doctors, nurses, other support services) diversity
Lack of leadership diversity	Leadership diversity
Language barriers (e.g., low number of bilingual staff or interpreter services)	Language (e.g., high number of bilingual staff or interpreter services)
Lack of prioritization of culturally responsive health communication (e.g., lack of inclusive language)	Prioritization of culturally responsive health communication (e.g., presence of inclusive language)
Lack of patient trust (in institution/staff/services)	Patient trust (in institution/staff/services)
N/A	N/A
7. Culture of change domain	7. Culture of change domain
Lack of culture of/openness to change	Culture of/openness to change
Missing previous history of quality improvement/protocol implementation	Previous history of quality improvement/protocol implementation
Lack of data to show progress/change	Presence of data to show progress/change
Poor work group norms (e.g., missing/no opportunities for constructive feedback)	Functional work group norms (e.g., opportunities for constructive feedback)
Limited organizational health literacy (e.g., lack of focus on improving health literacy)	High organizational health literacy (e.g., organization focuses on improving health literacy)
N/A	N/A
8. Please rank from 1-7 (in order) how you would prioritize addressing these BARRIER DOMAINS to achieve equitable care at your center. (1 being first priority, 7 being last priority)	8. Please rank 1-7 (in order) how you would prioritize enhancing these FACILITATOR DOMAINS to achieve equitable care at your center. (1 being first priority, 7 being last priority)
Organizational and Structural	Organizational and Structural
Knowledge and Resources	Knowledge and Resources
Cost and Supply Constraints	Cost and Supply
Time Constraints	Time Efficiencies
Staffing	Staffing
Culturally Relevant Factors	Culturally Relevant Factors
Culture of Change	Culture of Change
9. Could you explain why you ranked these BARRIER DOMAINS in this order?	9. Could you explain why you ranked these FACILITATOR DOMAINS in this order?
10. Were there other SPECIFIC BARRIERS missing that you wanted to bring to our attention?	10. Were there any SPECIFIC FACILITATORS missing that you wanted to bring to our attention?

Outcomes

The main outcomes were perceived barriers and facilitators to healthcare equity in pediatric TBI care during hospitalization: (1) across all PTCs and (2) between Level I and II PTCs. We also elicited recommendations on the prioritization of barrier and facilitator domains from Level I and II PTCs to achieve acute healthcare equity.

Analysis

Descriptive statistics were used to describe the following survey data in total and by level of PTC: (1) site organizational characteristics, including pediatric admissions, staffing, certification, and availability of staff; (2) TBI champion perceptions of leading barrier domains and associated specific barriers; (3) TBI champion perceptions of leading facilitator domains and associated specific facilitators; (4) prioritization weighted ranking scores to address barrier and facilitator domains; and (5) free text responses.

To determine leading barrier and facilitator domains, we defined “majority agreement” as at least 50% consensus on the selection of specific barriers and facilitators: (1) among all PTCs and (2) between Level I and II PTCs separately. Prioritization weighted ranking scores for addressing barrier and facilitator domains were calculated for each of the seven domains overall and by level of PTC, using a weighted prioritization matrix scoring method [25], in which the study team assigned a weighted score to each ranking from 1 to 7. Responses that ranked a domain as the first priority were multiplied by seven; and each subsequent priority was multiplied by a weight that decreased by one from the previous priority. Once weighted scores were calculated for each priority, scores were summed across each domain to produce a final weighted prioritization score for each barrier and facilitator domain [25]. The concurrent nested design of free-text responses was used to provide additional context to the quantitative survey responses by examining reasons for priority rankings of barrier and facilitator domains and identifying additional specific barriers and facilitators [26].

## Results

Participating sites

One of the invited 11 PTCs withdrew after expressing initial interest, leaving a final sample of 10 geographically and racially/ethnically diverse PTCs (91% of invitees participated and 100% survey response rate among participants) between September 2022 and August 2023. Eight of 10 PTCs reported affiliation with an adult trauma center.

The six Level I PTCs were as follows: Arkansas Children’s Hospital (South-West South Central), Children’s Hospital of Pittsburgh-University of Pennsylvania Medical Center (Northeast-Middle Atlantic), C.S. Mott Children’s Hospital-University of Michigan Health System (Midwest-East North Central), Johns Hopkins Children’s Center-Johns Hopkins Medicine (South-South Atlantic), Texas Children’s Hospital (South-West South Central), and Maria Fareri Children’s Hospital-Westchester Medical Center Health Network, Valhalla, NY (Northeast-Middle Atlantic).

The four Level II PTCs were as follows: Children’s Hospital and Medical Center Nebraska (Midwest-West North Central), Harbor-University of California Los Angeles Medical Center (West-Pacific), Mary Bridge Children’s Hospital, Tacoma, WA (West-Pacific), and St. Luke’s Children’s Hospital, Boise, ID (West-Mountain).

Organizational characteristics

**Figure 1:**
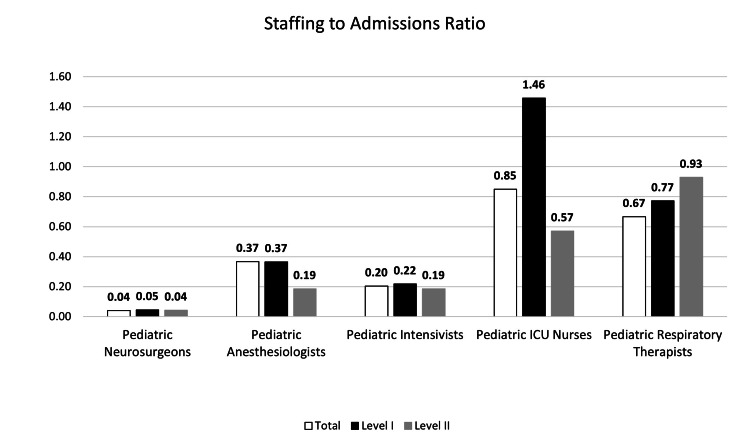
Staffing-to-admissions ratio patterns of 10 participating pediatric trauma centers by trauma center designation ICU: intensive care unit

**Figure 2:**
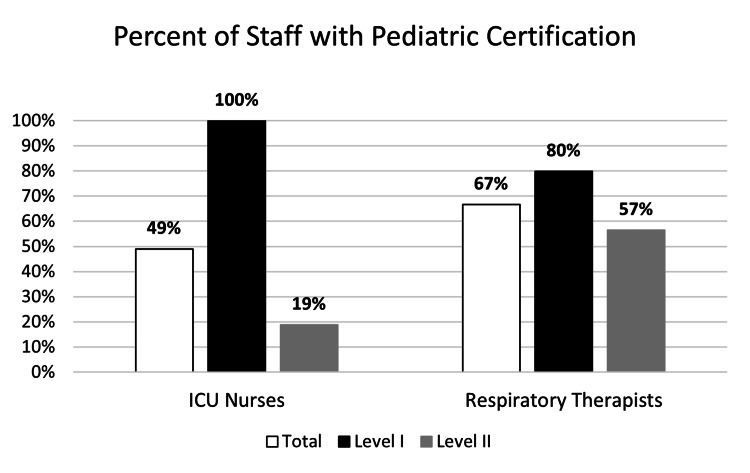
Staffing pediatric certification patterns of 10 participating pediatric trauma centers by trauma center designation ICU: intensive care unit

**Figure 3:**
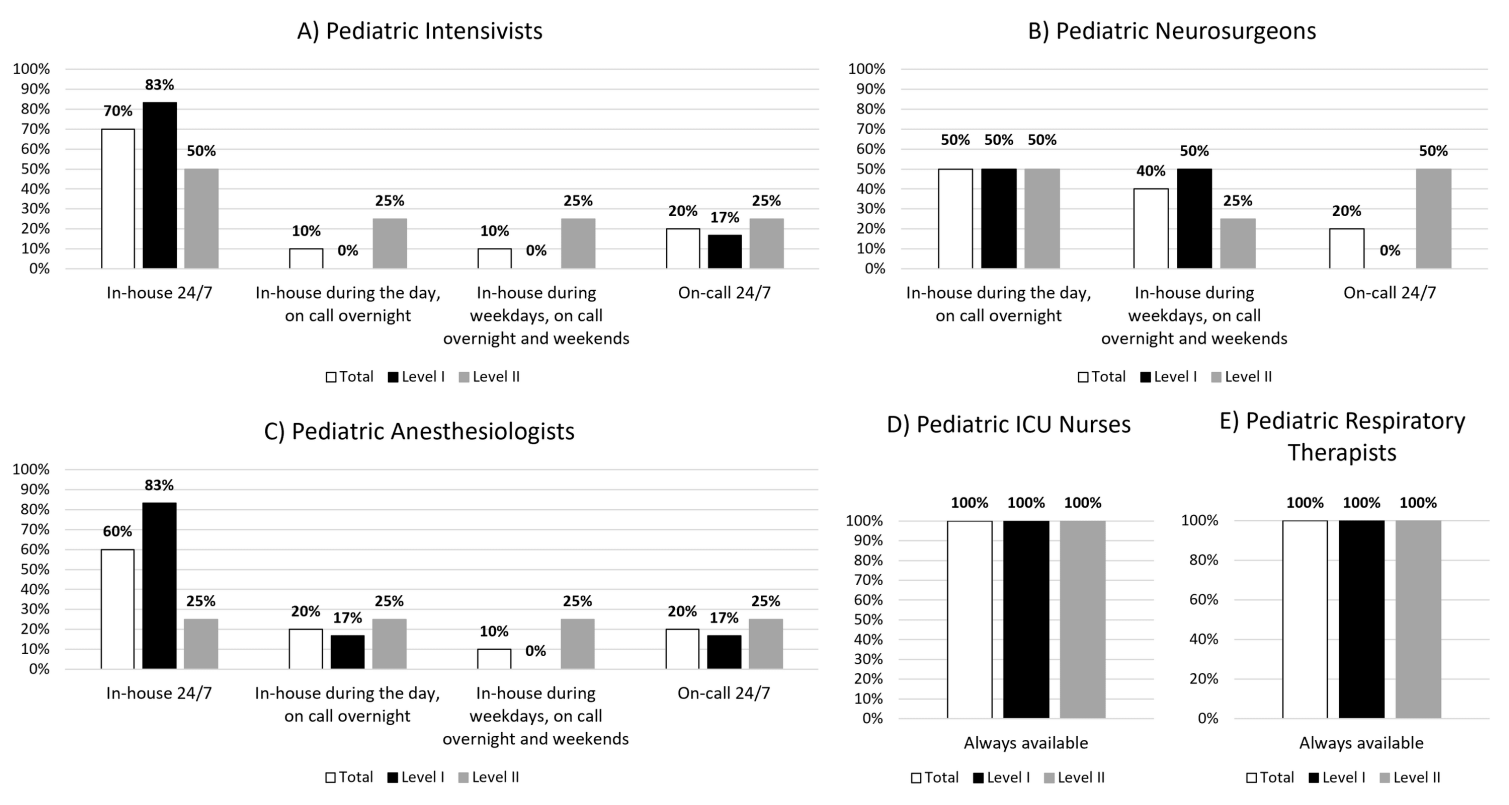
Staffing availability patterns of 10 participating pediatric trauma centers by trauma center designation Note: Sites were allowed more than one selection for staff availability in the survey. As a result, the sum of percentages across categories can be greater than 100% ICU: intensive care unit

Organizational characteristics are presented in Table 2 and Figures 1-3. Level I PTCs reported more median emergency department beds (40.5 vs. 23) and pediatric inpatient beds (254.5 vs. 98 beds), higher median upper-age limit for pediatric admissions (21 vs. 18 years), more median pediatric ICU nursing staff (128 vs. 20), and more median annual TBI admissions (88 vs. 35 patients/year) compared to Level II PTCs. Level II PTCs reported receiving more median TBI transfers from other hospitals than Level I PTCs (23 vs. 14 patients/year). Level I PTCs reported more nursing and respiratory therapy staff with a pediatric certification (100% nursing and 80% respiratory therapists) than Level 2 PTCs (19% nursing and 57% respiratory therapists).

**Table 2:** Organizational characteristics of 10 participating pediatric trauma centers *Note: There is one response missing for transfers, Level I ED: emergency department; ICU: intensive care unit; TBI: traumatic brain injury

Hospital	Total (n=10)	Level I pediatric trauma center (n=6)	Level II pediatric trauma center (n=4)
Affiliated with Level I adult trauma center, n (%)	8 (80%)	6 (100%)	2 (50%)
Pediatric inpatient beds, median (range)	195.5 (36-750)	254.5 (136-750)	98 (36-225)
Upper age limit for children, years, median (range)	19.5 (14-26)	21 (14-26)	18 (18-21)
Annual pediatric trauma admissions, median (range)	613 (217-1504)	803 (340-1504)	449.5 (217-830)
Number of computed tomography scanners, median (range)	3 (2-5)	3.5 (2-5)	3 (3-4)
Number of magnetic resonance scanners, median (range)	3.5 (2-8)	4 (3-8)	2.5 (2-3)
Number of operating rooms, median (range)	17.5 (10-62)	23 (14-62)	16.5 (10-18)
ICU			
Total pediatric ICU beds, median (range)	27 (8-130)	29 (18-130)	17 (8-130)
Annual pediatric TBI admissions, median (range)	73.5 (20-158)	87.5 (20-158)	35 (24-105)
Annual pediatric TBI admissions transferred from other hospitals*, median (range)	15.5 (2-105)	14 (2-35)	22.75 (15-105)
Number of attending pediatric neurosurgeons, median (range)	3 (1-6)	4 (2-6)	1.5 (1-3)
Number of attending pediatric anesthesiologists, median (range)	27 (2-91)	32 (4-91)	6.5 (2-29)
Number of attending pediatric intensivists, median (range)	15 (4.5-79)	19 (10-79)	6.5 (4.5-20)
Number of ICU nurses, median (range)	127.5 (20-270)	127.5 (81-270)	106 (20-210)
Number of pediatric-certified ICU nurses, median (range)	62.5 (16-270)	127.5 (40.5-270)	20 (16-74)
Number of respiratory therapists, median (range)	73.5 (12-260)	84.5 (20-260)	57.5 (12-86)
Number of pediatric respiratory therapists, median (range)	49 (2-260)	67.5 (10-260)	32.5 (2-50)
Nurse to pediatric ICU patient ratio	(1:3-2:1)	(1:3-2:1)	(1:2-2:1)
ED			
Number of ED beds designated for pediatric patients, median (range)	31.5 (6-53)	40.5 (20-53)	23 (6-33)
Pediatric-specific (Broselow) cart present in ED, n (%)	10 (100%)	6 (100%)	4 (100%)

Pediatric TBI champions

Physician disciplines represented were as follows: surgery (n=5), critical care medicine (n=2), anesthesiology (n=1), neurology (n=1), and neurosurgery (n=1).

Perceived barriers and facilitators to the provision of equitable inpatient pediatric TBI care

Barrier Domains and Associated Specific Barriers Across 10 Participating PTCs

**Figure 4:**
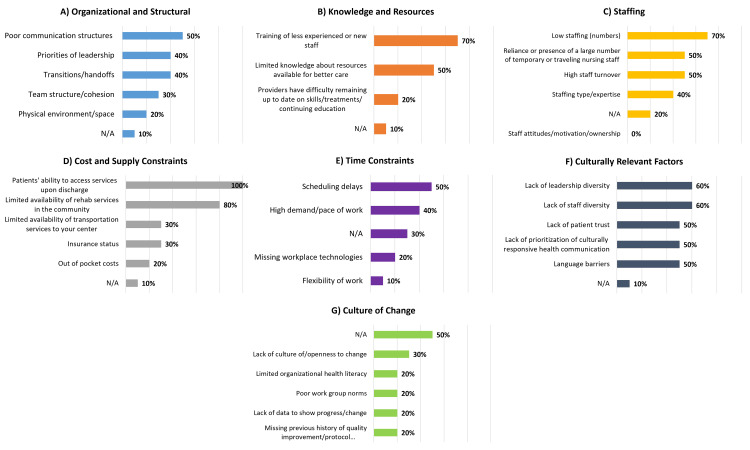
Physicians' perceptions of barriers to equitable acute pediatric TBI care across 10 pediatric trauma centers by barrier domain TBI: traumatic brain injury

The three domains associated with the most frequently selected specific barriers were cost and supply constraints (100% selected "lack of access to services upon discharge"; 80% selected "limited availability of rehab services in the community"), knowledge and resources (70% selected "lack of training of less experienced or new staff"), and staffing (70% selected "inadequate staffing") (Figure 4). The three barrier domains with the fewest selections of associated specific barriers were: culture of change (30% selected "lack of culture of openness of change"), time constraints (50% selected "scheduling delays"), and organizational and structural (50% selected "poor communication structures").

Majority Agreement on Specific Barriers Across Domains Among and Between Level I and Level II PTCs

**Figure 5:**
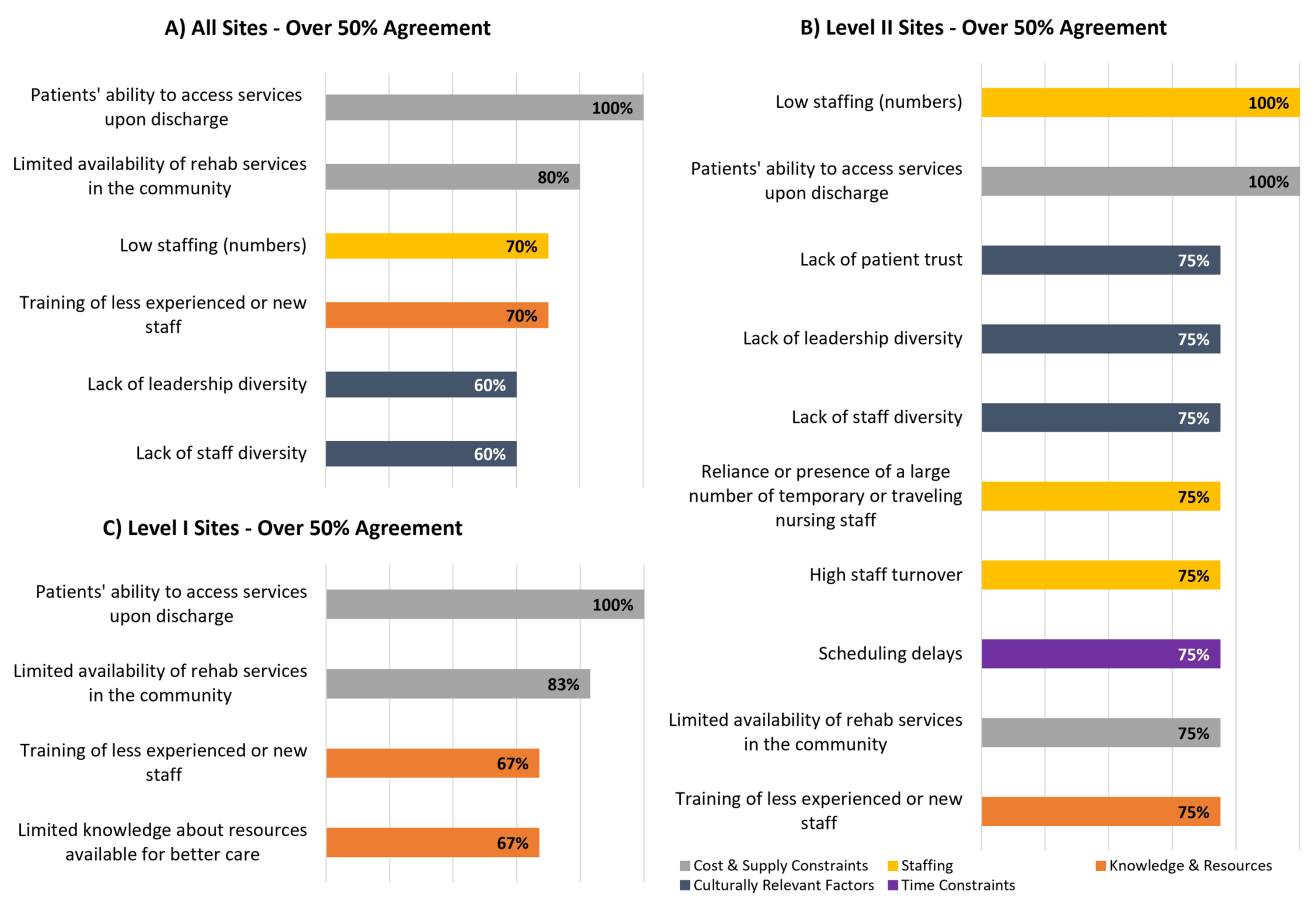
Majority agreement on physicians' perceptions of barriers to equitable acute pediatric TBI care across 10 pediatric trauma centers by trauma center designation and domain Domains shown: cost and supply constraints (patients' ability to access services upon discharge; limited availability of rehab services in the community); staffing (low staffing numbers; reliance or presence of a large number of temporary or traveling nursing staff; high staff turnover; knowledge and resources (training of less experienced or new staff; limited knowledge about resources available for better care); culturally relevant factors (lack of leadership diversity; lack of staff diversity; lack of patient trust); time constraints (scheduling delays) TBI: traumatic brain injury

Collectively, Level I PTCs reported four leading barriers: lack of access to services upon discharge (100%), limited availability of community rehabilitation services (selected by 83%), lack of training of less experienced or new staff (selected by 67%), and limited knowledge of resources available for better care (selected by 67%) (Figure 5).

Level II PTCs selected the following leading barriers: (1) low staffing numbers (100%), (2) inability to access services upon discharge (100%), and (3) an eight-way tie (75% selected): lack of trust between patients and providers, lack of leadership diversity, lack of staff diversity, high reliance on temporary staff, high staff turnover, scheduling delays, limited availability of community rehabilitation services, and lack of training of staff.

Majority Agreement on Specific Barriers Between Level I and Level II PTCs

Three of four barriers collectively selected by Level I PTCs were also identified as barriers by Level II PTCs and most identified barriers were reported by both Level I and II PTCs (Figure 5). Limited knowledge of resources was uniquely identified by Level I PTCs. Level I and II PTCs reported the same leading barrier (‘access to services upon discharge’). Staff attitude and motivation were not reported as a barrier by any centers.

Facilitator Domains and Associated Specific Facilitators Across 10 Participating PTCs

**Figure 6:**
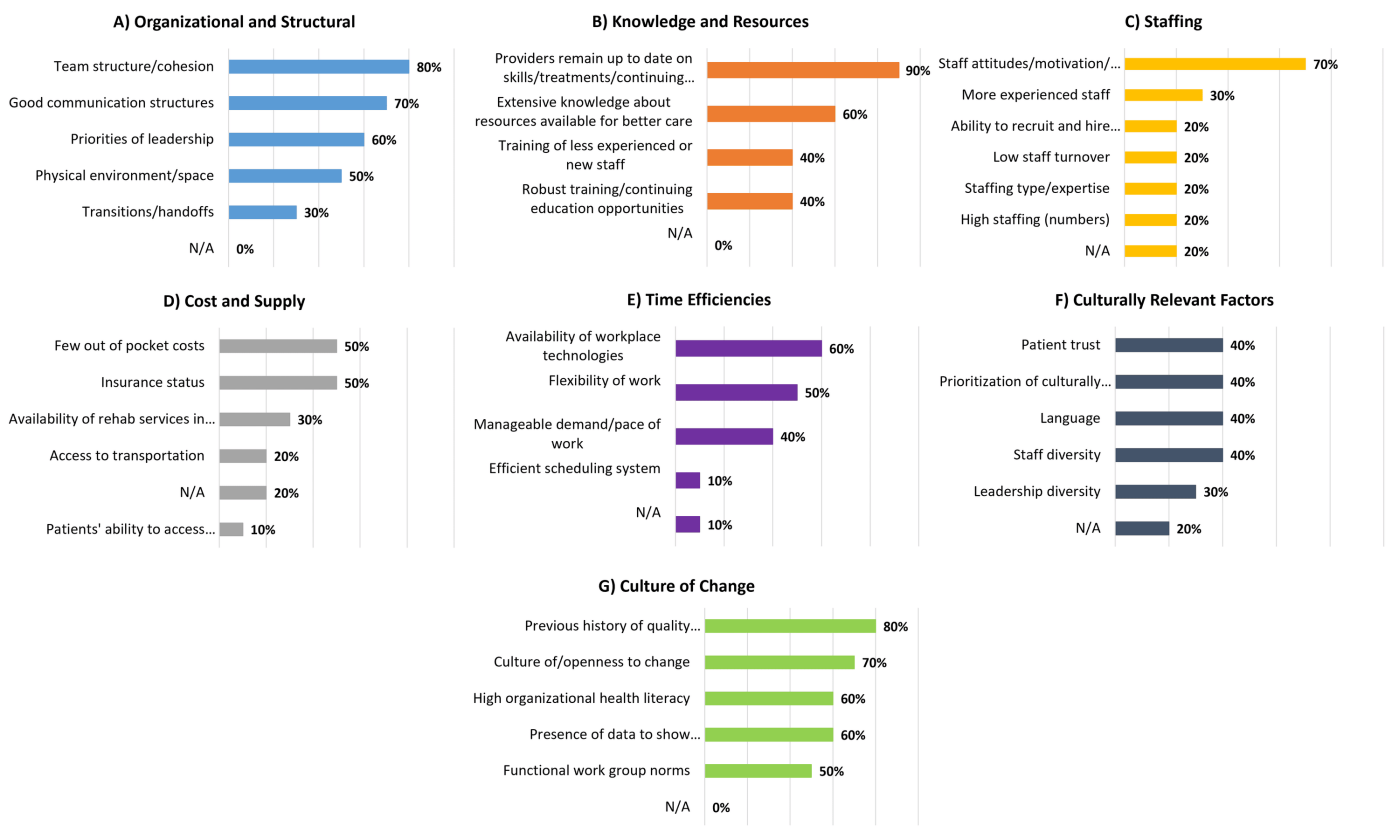
Physicians' perception of facilitators to equitable acute pediatric TBI care across pediatric trauma centers by facilitator domain TBI: traumatic brain injury

Three leading facilitator domains with the most frequently selected associated specific facilitators were knowledge and resources (90% selected "providers remain up to date on skills, treatments, and continuing education"), organizational and structural (80% selected "team structure and cohesion"), and culture of change (80% selected "previous history of quality improvement and protocol implementation") (Figure 6). The three facilitator domains with the fewest selections of associated specific facilitators were time efficiencies (60% selected "availability of workplace technologies"), cost and supply (50% selected "few out-of-pocket costs" and "insurance status"), and culturally relevant factors (40% selected "patient trust", "language", "staff diversity", and "prioritization of culturally responsive health communication").

Majority Agreement on Specific Facilitators Across Domains Among and Between Level I and Level II PTCs

**Figure 7:**
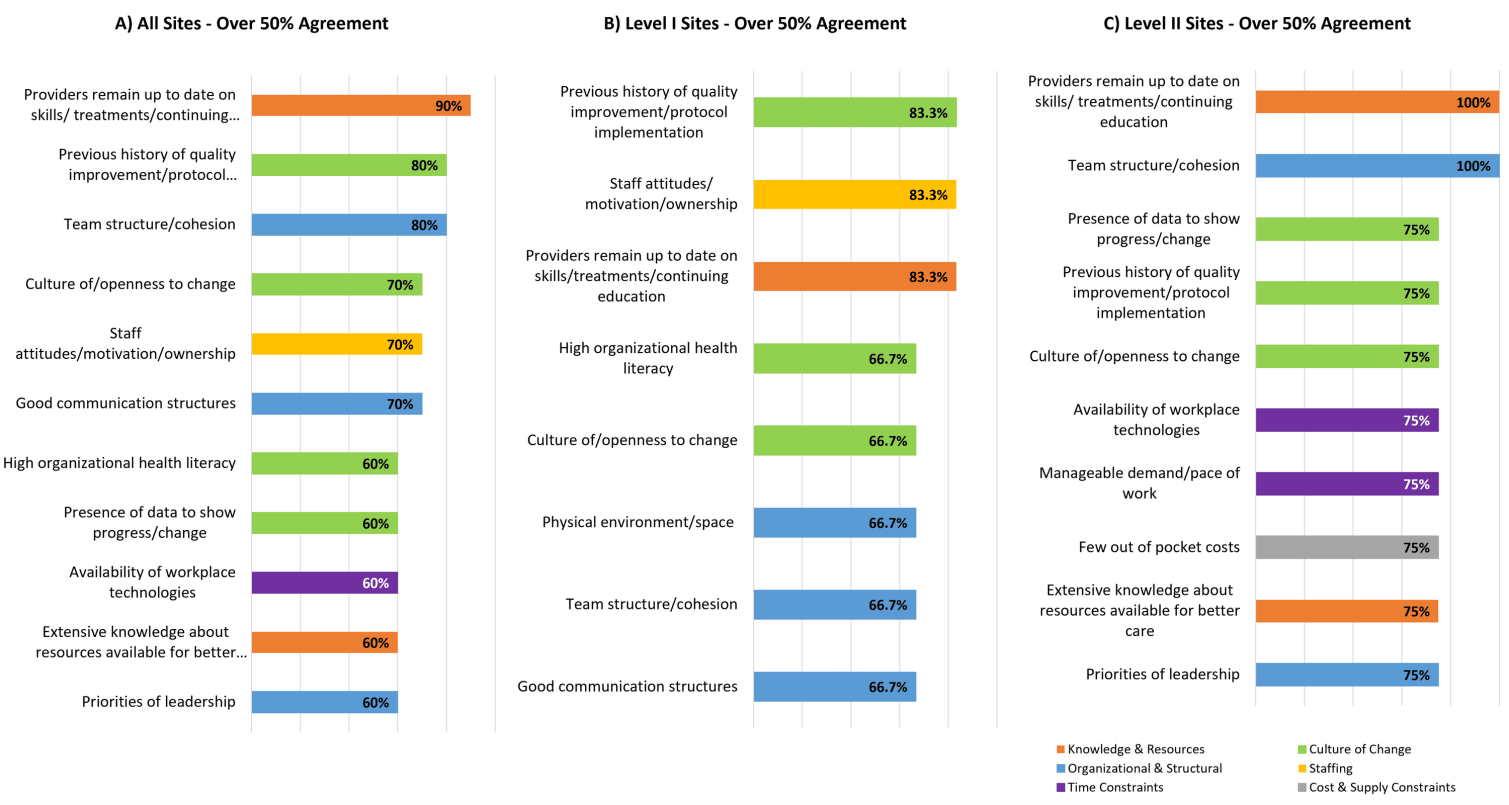
Majority agreement on physicians' perception of facilitators to equitable acute pediatric TBI care across pediatric trauma centers by trauma center designation and domain Domains shown: knowledge & resources (providers remain up to date on skills/treatments/continuing education; extensive knowledge about resources available for better care); culture of change (previous history of quality improvement/protocol implementation; culture of/openness to change; high organizational health literacy; the presence of data to show progress/change); organizational & structural (team structure/cohesion; good communication structures; priorities of leadership; physical environment/space); staffing (staff attitudes/motivation/ownership); time efficiencies (availability of workplace technologies; manageable demand/pace of work); cost & supply (few out-of-pocket costs) TBI: traumatic brain injury

Level I PTCs selected eight total specific facilitators with majority agreement: (1) a three-way tie (83.3% selected) for the history of quality improvement and protocol implementation, staff attitudes/motivation/ownership, and providers remain up to date on skills/treatments and continuing education, and (2) a five-way tie (66.7% selected) for high organizational health literacy, culture of openness to change, physical environment/space, team structure/cohesion, and good communication structures (Figure 7).

Level II PTCs reported 10 specific facilitators with majority agreement: (1) a two-way tie (100%) for providers to remain up to date on skills, treatments/continuing education, and team structure and cohesion, and (2) an eight-way tie (75% selected) for presence of data to show progress/change, previous history of quality improvement/protocol implementation, culture of openness to change, availability of workplace technologies, manageable pace of work, limited out-of-pocket costs, provider knowledge about resources for better care, and priorities of leadership.

Majority Agreement on Specific Facilitators Between Level I and Level II PTCs

All facilitators with majority agreement received at least one selection from a Level I or II PTC, and nine sites (90%) reported provider skills and education as facilitators. However, four of the eight leading facilitators that Level I PTCs identified were not identified collectively as leading facilitators by Level II PTCs (Figure 7).

Priorities for Addressing Barriers and Facilitators Across and by PTC Level

**Figure 8:**
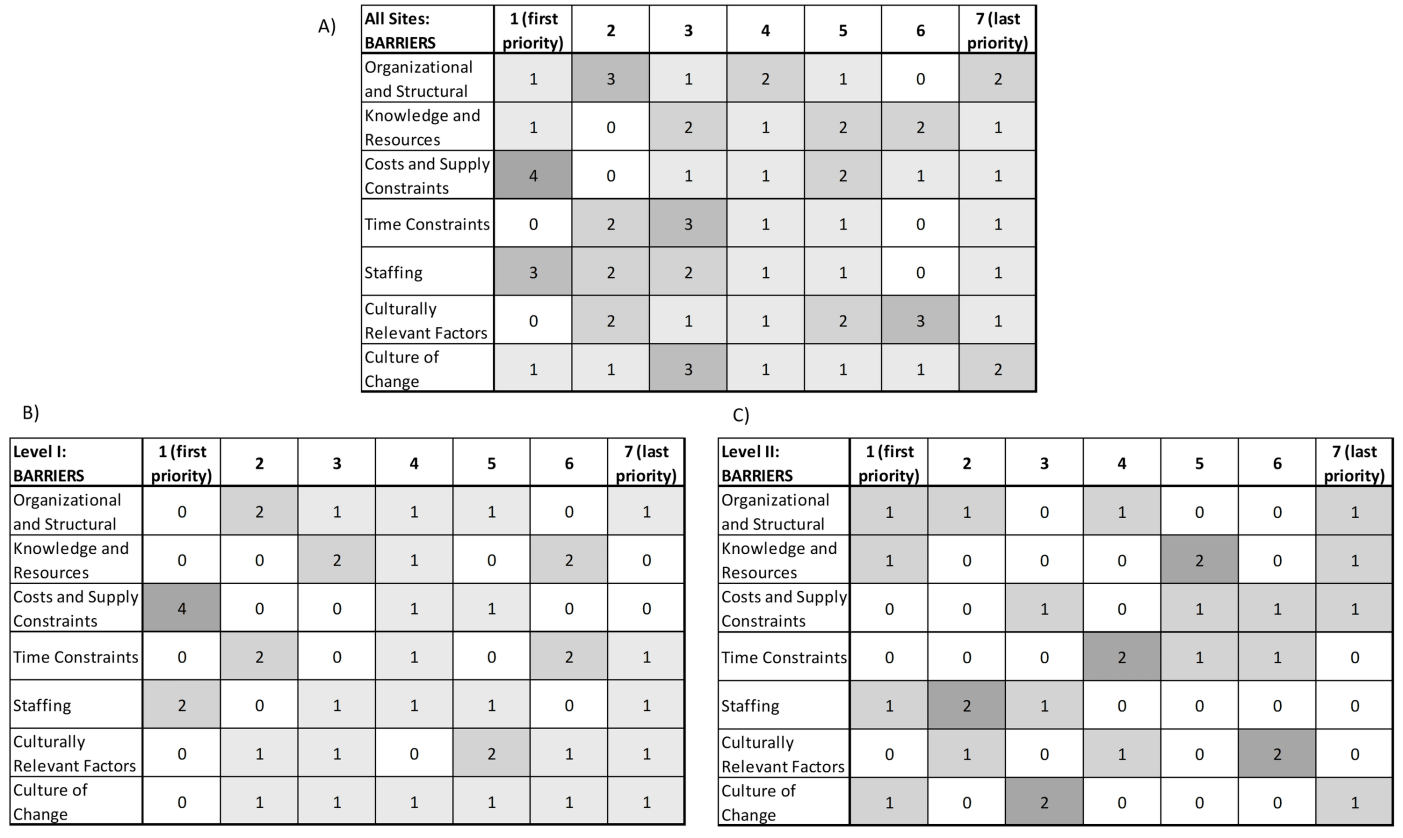
Raw data and prioritization matrix for redressing barrier domain in provision of equity in acute pediatric TBI care Note: There is one response missing for knowledge and resources, Level I: barriers TBI: traumatic brain injury

**Figure 9:**
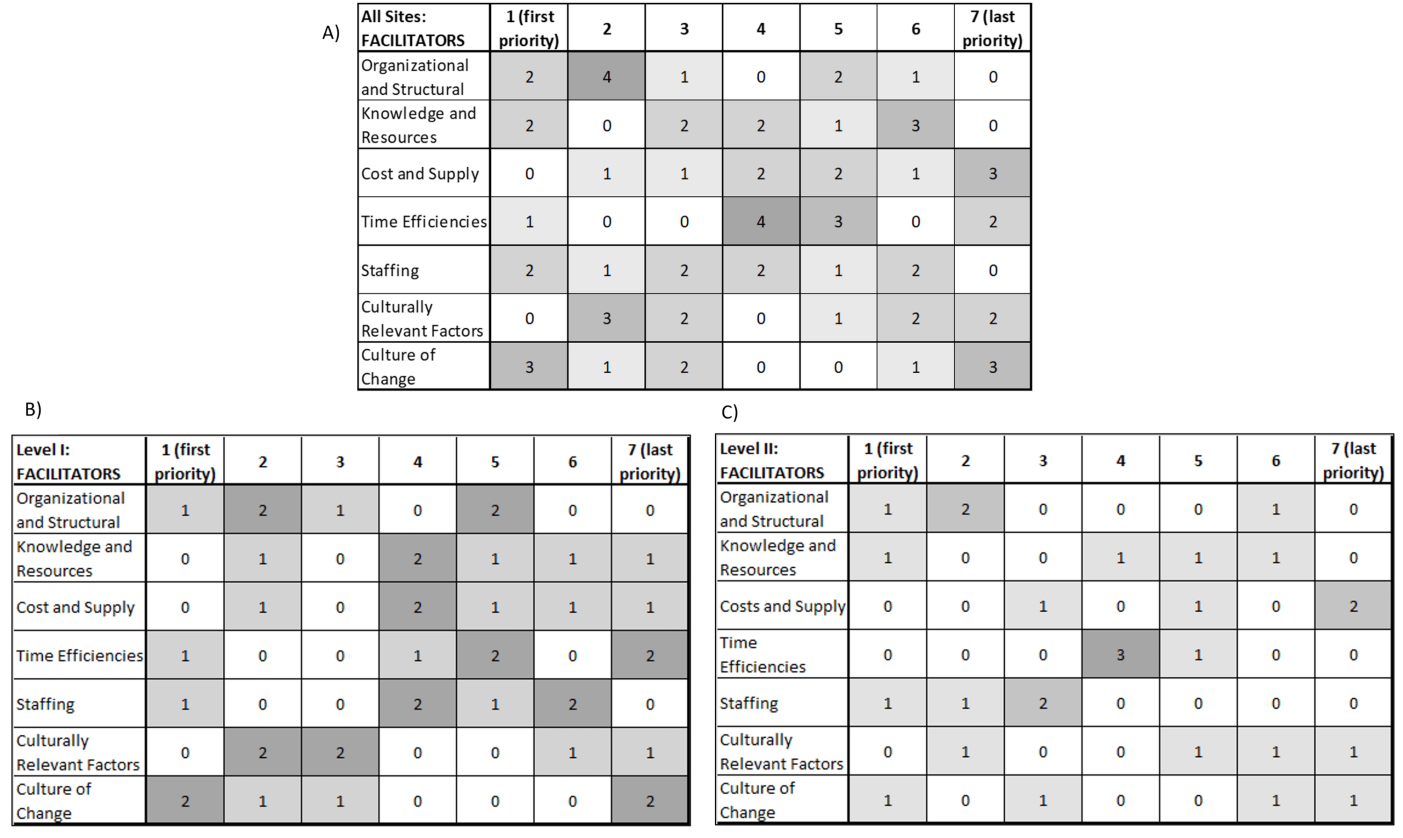
Raw data and prioritization matrix for supporting facilitator domains in provision of equity in acute pediatric TBI care TBI: traumatic brain injury

Raw data and prioritization matrices for addressing barriers and facilitators are shown in Figures 8-9. Table 3 shows the results of our weighted scoring method: the highest priority barrier domain was staffing, followed by cost and supply constraints, and organizational and structural factors. The highest priority facilitator domain was the organizational and structural, followed by staffing and culture of change. Staffing and organizational and structural were leading priorities for both barriers and facilitators, followed by culture of change.

**Table 3:** Staffing availability patterns of 10 participating pediatric trauma centers by trauma center designation Note: Sites were allowed more than one selection for staff availability in the survey. As a result, the sum of percentages across categories can be greater than 100% ICU: intensive care unit

Rank	Barriers			Facilitators		
	Total	Level I	Level II	Total	Level I	Level II
1	Staffing	Cost and supply constraints	Staffing	Organizational and structural	Organizational and structural	Staffing
2	Cost and supply constraints	Staffing	Organizational and structural (tie 1)	Staffing	Culture of change	Organizational and structural
3	Organizational and structural	Organizational and structural	Culture of change (tie 1)	Culture of change	Culturally relevant factors	Knowledge and resources
4	Culture of change	Time constraints (tie)	Knowledge and resources (tie 2)	Knowledge and resources	Staffing	Time efficiencies (tie)
5	Time constraints	Culture of change (tie)	Culturally relevant factors (tie 2)	Culturally relevant factors	Knowledge and resources (tie)	Culture of change (tie)
6	Culturally relevant factors	Culturally relevant factors	Time constraints	Time efficiencies	Cost and supply (tie)	Culturally relevant factors
7	Knowledge and resources	Knowledge and resources	Cost and supply constraints	Cost and supply	Time efficiencies	Cost and supply

Concurrent nested analysis to augment understanding of quantitative survey responses

Reasons for Priority Domain Rankings

Free text responses identified decisions and rationale for priority ranking of barriers and facilitator domains (Table 4). Explanations for barrier rankings included staffing shortages, costs, and desire for a culture of change but other barriers impeded cultural adaptations and acceptance.

**Table 4:** Verbatim free text responses identified the decisions and rationale for priority ranking of barriers and facilitator domains Peds: pediatric; PICU: pediatric intensive care unit; RT: resident trainee; TBI: traumatic brain injury

Barriers		Facilitators	
Could you explain why you ranked these barriers in this order?	Were there other barriers missing that you wanted to bring to our attention?	Could you explain why you ranked these facilitators in this order?	Were there any facilitators missing that you wanted to bring to our attention?
The system is primed for cultural change; we are all burned out, exhausted, overworked, and understaffed	In-person translators	Staffing trumps all	Access to social workers and care coordinators
The main issue we've observed is racial/economic barriers to access to care. Additionally, this center has distinct racial differences in the mechanism of injury (i.e., Black children are more likely to have sustained penetrating trauma)	Trainee (resident/fellow) diversity	Enhancing time, staff, and supply would seem to help this patient population achieve equity. While the other factors are important, we feel these have already been addressed at our facility	We have surgical intensivists rounding in the PICU
Primarily, staffing shortages have become a significant consideration at our institution as many urgent cases are delayed. This has significant adverse effects on patient outcomes	Overall county priorities/funding often inhibit our organization from making the changes we know need to be made	I ranked the areas that need more enhancement/improvement highly to supplement our already strong qualities	
I think the barriers listed at the top will have the most impact on being able to address the lower-ranked barriers	The lack of diversity in my area is an issue	Having knowledge and resources will assist our facilitators	
I think working on staffing issues and staff knowledge will have the biggest impact	In our institution, there is a historical lack of trust in the medical school and hospital by the surrounding local community. So this creates some barriers	A tangible "why" aligns the organization and levels the culture which attracts altruistic individuals to build and deliver the services needed	
Culture promotes teamwork, innovation, and transformation. It is the foundation of the organization and sets the aspirations and vision	Implicit biases of providers can be a barrier to equitable care. Additionally, self-fulfilling prophecy and experience bias are barriers to care (aka PICU providers that only see the worse outcomes return may have a bias in outcomes which can affect care compared to providers that actually follow all patients long term). Community resources and support services; especially for young children with TBI (teens can often access adult resources but infants, toddlers, and young school age require Peds-specific services which are harder to find	The institution (hospital, university) has a focus on change, culturally relevant factors and diversity. There is generally a depth of resources, but staffing (especially at the nursing & RT level) has created some barriers	
Our organization seems generally open to a culture of change and often focused on culturally relevant factors. However, we seem to often be limited by costs/financial resources, and staffing issues have definitely negatively impacted things	My interest in being a TBI champion in my role… to understand best practices and develop a framework for implementing and tracking improvement	Under new leadership, TBI care is much more highly prioritized at our institute. As a very diverse city, culturally relevant factors are critical, and a culture of change (new and improved care; rethinking current ways). However, addressing cost/supply is then critical to facilitating equitable care	
I feel that without organizational and cost/supply constraints addressed, there is less to work with but then culturally relevant factors must be one of the top 3 priorities. staffing is critical (but without the other 3 in place, then staffing will matter less - you can have people but without the more global support, the number of staff will matter less); finally, I feel that knowledge and resources are something more easily taught/remedied and then time constraints will improve inevitably with all of the other barriers addressed		There is high priority and value placed on equity. Increasing resources are being provided. The organization has always valued outcome improvement and as important leadership in driving regional and national change	
We are a referral center for the state and the majority of our severe trauma is referred here. Care and follow-up after discharge are a problem. We collect and track, but have not been focusing on the data to track improvement in our TBI management. That plays into knowledge and resources. Transitions in care and plans are not smooth and could be improved but that is a lower priority. Staffing and training are not fundamental issues for us as we have adequate resources

“...barriers listed at top will have the most impact on being able to address the lower ranked barriers.​”

“The system is primed for cultural change; [but] we are all burned out, exhausted, overworked, and understaffed.”

“Our organization seems generally open to a culture of change, and often focused on culturally relevant factors. However, ...often be limited by costs/financial resources, and staffing issues have... negatively impacted things.”

TBI champions identified where healthcare is equitable for patients and families while acknowledging that continued change must occur.

“Enhancing time, staff, and supply would seem to help this patient population achieve equity. While the other factors are important, we feel these have already been addressed at our facility.”

Identification of Additional Specific Barriers and Facilitators From Free Text

Level II PTCs additionally identified county priorities/funding and a lack of diversity around center location as barriers, while Level I PTCs mentioned provider implicit biases as a barrier and suggested access to social workers and care coordinators and team structure and cohesion with specialists rounding in the ICU as facilitators.

## Discussion

This study's goal was to understand physician champion perceptions of barriers and facilitators to achieving healthcare equity for children hospitalized with TBI. The main findings from our sample of Level I and II PTC physician champions are as follows: (1) Level I and II PTC respondents endorsed some common barriers and facilitators, but leading barriers and facilitators varied between Level I and II PTCs, and (2) Level I and II PTCs reported different high priority actions to achieve equity for children hospitalized with TBI. This is the first report examining physician TBI champion perspectives on equity in TBI care across geographically diverse centers, including rural and Level II PTC perspectives. Our findings suggest opportunities for health systems to share learnings among and between Level I and II PTCs to benefit patients and to develop local protocols with tailored roadmaps providing equitable patient and family-centered care for children hospitalized with TBI.

The Brain Trauma Foundation (BTF) guidelines provide best practices based on published evidence for acute management of critically ill children and adolescents with TBI [13,27-28], and adherence to BTF recommendations during hospitalization is associated with improved survival and favorable discharge disposition [29-30]. While achieving adherence to evidence-based guidelines improves outcomes, adherence to guidelines alone does not guarantee equity in healthcare delivery or outcomes. In fact, quality improvement initiatives without an explicit focus on equitable implementation may worsen existing disparities [31-32]. The BTF guidelines do not address goals or metrics for assuring equity in pediatric TBI care delivery, but the National Academy of Sciences Engineering and Medicine task force recently identified equity as a high priority in improving acute TBI care, rehabilitation care, and outcomes for adults and children with TBI [33-34]. An important step to address this call to action and gaps in knowledge, research, and practice is to understand the experiences of clinicians with expertise in TBI care.

Our work sheds light on perceptions of barriers and facilitators to inform the development of metrics and pathways to reduce healthcare disparities in pediatric TBI care [35]. While the sample is small and responses were gathered only from physician TBI champions, the TBI champions represented various specialties, and our inclusion of geographically diverse centers helped us understand the needs of diverse populations cared for at these centers. Although sites were initially contacted based on known professional networks, physician champions were identified based on prespecified criteria (leadership, scholarly activity, research expertise, and/or educational program development in TBI) to respond to the survey and serve as leadership points of contact in further equity improvement initiatives by our research group.

Given their roles, physician champions were assumed to have knowledge about their institutions, relationships with other physicians and other healthcare disciplines, and involvement in hospital-level decision-making regarding TBI management, allowing them to provide unique expertise not only based on their own clinical experiences but also system-level factors. Our future work will investigate the experiences of other healthcare professionals to ultimately design and implement strategic initiatives to improve equity, including an examination of how longer LOS compounds risks of inequities experienced by patients awaiting post-discharge placement.

Similar to prior work [36-38], a leading specific barrier reported across participating Level I and II PTCs was a lack of access to post-discharge services, particularly rehabilitation care, contributing to disparities in long-term functional TBI outcomes among children from racial and ethnic minority groups [39-40]. Most TBI champions ranked time constraints and knowledge and resources as the lowest barrier domains and also reported a culture of change as a relatively low barrier domain potentially signaling organizational motivation and readiness to develop and implement interventions facilitating the delivery of equitable healthcare in TBI. Examples of organizational interventions include training to reduce implicit bias and/or practices that highlight healthcare disparities, addressing staffing deficits, and evaluating impacts of cost and supply constraints, cited as leading barriers across all PTCs but may be receptive to change. An economic analysis relating organizational characteristics (i.e. county hospital status) with supply and cost was beyond the scope of this work but is important.

Level II PTCs had more variability in agreement regarding leading specific barriers including lack of trust between patients and providers, lack of leadership diversity, lack of staff diversity, high reliance on temporary staff, high staff turnover, scheduling delays, limited availability of community rehabilitation services, and lack of staff training. Staffing shortages challenged the provision of high-quality patient-centered care, especially during the COVID-19 pandemic when healthcare and outcome disparities increased; this work likely reflects those experiences of TBI champions. While both Level I and II PTCs provide similar levels of clinical care, there may be differences in the availability of highly specialized subspecialists (typically surgical) and/or academic missions, potentially explaining higher pediatric-centered intensivist to admissions ratios in Level I than Level II PTCs, and a larger number of barriers reported by Level II PTCs. While we did not collect healthcare provider demographics at participating PTCs, increasing the diversity of the healthcare workforce is a strategy to build provider-patient trust and achieve favorable outcomes [41]. Future work is needed to develop strategies to reduce barriers at Level II PTCs, which care for a sizeable number of pediatric TBI admissions.

Participating PTCs provided a larger and more variable list of specific facilitating than barrier factors. Yet, nine of 10 sites reported provider skills and education as a facilitator. The fact that four of eight leading facilitators amongst Level I PTCs were not identified collectively as leading facilitators by Level II PTCs suggests that PTCs will need to develop local solutions despite many commonly identified barriers. We identified domains relevant to how we traditionally consider quality of care, and while we did not specifically ask this question, responses suggest that providing equitable care is important to providing quality care. Future work should consider integrating equity measures as part of quality improvement initiatives.

We solicited input to prioritize barrier and facilitator domains to conceive a roadmap for reducing in-patient TBI care disparities. Weighted responses reflect the ordering of priorities ranked by each site, with the highest priority (ranked #1) receiving the most weight and the lowest priority (ranked #7) receiving the least weight. Initially, we assumed that the least reported barrier domains and most reported facilitator domains would emerge as the highest recommended priority domain actions. However, respondents prioritized staffing and costs and supply constraints as leading addressable barrier domains while organizational factor domains and those related to developing a culture of change were leading facilitator domains. Although we cannot statistically compare differences in characteristics from our limited sample, we collected organizational information to present the context of findings, providing information for future work to understand organizational effects on the provision of TBI care. We speculate that the COVID-19 pandemic may have resulted in organizational culture emphasizing productivity, staffing, cost containment and supply chain constraints as barriers to consider in clinical care, as well as workflow flexibility and an important facilitator of quality clinical care.

Although our survey was informed by theoretical frameworks, we recognize that instrument bias may prevent an inclusive and complete list of barrier and facilitator domains as well as specific barriers and facilitators [42-44]. Therefore, we included a free text option, resulting in respondent identification of county priorities and funding, lack of diversity in the work location, and implicit biases of providers as additional barriers. Respondents identified access to social workers and care coordinators, team structure, and cohesion with specialists rounding in the ICU as promoting factors. Our data suggest that TBI champion prioritization and ranking of barriers and facilitators may need to be appraised with these additional considerations: 1) easiest to address (“lowest-hanging fruit”), 2) requiring the most time, resources, and intentional dedication to change, (“highest hurdle”) or 3) having the greatest margin for improvement (“most cost-effective”).

Future healthcare equity surveys should include free text responses from a larger pool of healthcare professionals, patients, and families for instrument refinement. Our prior work developed a family-centered care model and determined factors associated with provider adherence to severe pediatric TBI guidelines [20,29-30]. This was informed by families of children hospitalized with TBI. From this effort, we learned that three interrelated domains were associated with adherence to evidence-based medical guidelines: 1) perceived guideline credibility and applicability to individual patients; 2) implementation, dissemination, and enforcement strategies; and 3) provider culture, communication style, and attitude towards protocols. Similar to findings of common barrier and facilitator domains to delivery of evidence-based medical care, the present work adds to our understanding of overlapping domains and common approaches to achieving healthcare equity, resulting in an important opportunity to include high-priority equity measures in guideline-based inpatient TBI care.

Strengths of this study include an in-depth and novel examination of physician pediatric TBI champion perceptions, representation from geographically diverse PTCs, the inclusion of Level II PTCs which are not typically included in research, use of concurrent nested analyses to better understand prioritized solutions and inform the rationale of the domain prioritization rankings for both Level I and II PTCs. The main limitation is the small number of respondents and sample size number limiting generalizability to the range of PTCs, non-PTCs, and free-standing facilities not affiliated with adult trauma centers that provide inpatient care for children with TBI. This is pilot work and not all disciplines and healthcare worker voices were represented. Physician leader perceptions of barriers/facilitators may not necessarily correlate with the reality of barriers/facilitators contributing to healthcare disparities, but other studies have demonstrated that as direct providers of care, physicians can identify equity issues in their patient populations [45-46].

Some findings were not specific to equity in TBI care and may have reflected other reasons for perceived inequities. We did not examine how respondents made their determinations since the aim was to document their perceptions. Physician-leader perceptions may not correlate with other physician perceptions. Despite these limitations, identifying initially perceived barriers and facilitators to equitable pediatric TBI care offers an opportunity for further examination on addressing and understanding these findings in future work. We intentionally did not define “equitable” because the goal of this work was to contribute to the development of a clinically-informed working definition. In this study, we examined perceptions, and future work will examine patient experiences of inequities, allowing evaluation of alignment between perception and reality. Future work should also consider triangulation of provider perspectives and patient information to yield an equity roadmap for the care of children with TBI.

## Conclusions

This study offers new insights into the perceptions of physician TBI champions about leading barriers and facilitators related to healthcare equity during inpatient pediatric TBI care. While there are certain commonly perceived specific barriers between Level I and Level II PTCs, we found more variability in specific facilitators, suggesting the need for locally derived roadmaps for redressing barriers. The lack of alignment among priorities between barrier and facilitator domains suggests that organizational and structural factors are important contributors to pediatric TBI care delivery.
